# Peripheral Artery Disease Intervention: Drug-Coated Balloon vs Drug-Eluting Stent, A Long-Term Comparison

**DOI:** 10.1155/2022/5175607

**Published:** 2022-08-27

**Authors:** Nathan Marzlin, M. Fuad Jan, Louie Kostopoulos, Ana Cristina Perez Moreno, Tanvir Bajwa, Suhail Q. Allaqaband

**Affiliations:** ^1^Aurora Cardiovascular and Thoracic Services, Aurora Sinai/Aurora St. Luke's Medical Centers, University of Wisconsin School of Medicine and Public Health, 2801 W. Kinnickinnic River Parkway, Milwaukee, WI 53215, USA; ^2^Cardiovascular Research, Advocate Aurora Research, Advocate Aurora Health, 960 N. 12th St., Ste. 4120, Milwaukee, WI 53233, USA

## Abstract

**Objectives:**

The aim of the study is to evaluate current trends and long-term durability of both drug-eluting stents (DES) and drug-coated balloons (DCB) in the treatment of peripheral artery disease (PAD).

**Background:**

PAD affects more than 200 million people worldwide. Endovascular treatment of critical PAD has advanced in recent years. DES and DCB have demonstrated superiority compared to balloon angioplasty or bare metal stenting. The current literature lacks any long-term, direct comparison.

**Methods:**

A retrospective analysis was completed on patients who had femoral-popliteal interventions from June 2014 to June 2018 with either DCB or DES. Patient medical data and lesion characteristics were retrieved using the Vascular Quality Initiative database. Outcomes were analyzed through December 2019. Primary endpoint of time to clinical event-driven target lesion reintervention (TLR) and secondary endpoint of all-cause mortality were examined.

**Results:**

Four hundred eighty-three patients with a total of 563 interventions met the inclusion criteria. Three hundred fifty-nine DCB and 204 DES were performed. Of the DCBs, 132 required bailout stenting at the time of procedure. The mean time for TLR in the DES group was 1,277 days (SD 546), compared to 904 days (SD 330.1) for DCB. For patients requiring TLR, DES remained patent significantly longer (373 days longer on average) (*p* < 0.001). For all-cause mortality there was no significant difference at 50 months between DCB and DES (*p* = 0.06).

**Conclusions:**

In patients who required TLR, DES had a significantly longer length of time to reintervention vs DCB (average 373 days), although no difference in mortality was observed.

## 1. Introduction

Endovascular treatment of peripheral artery disease (PAD) has become an important part of current medical practice. With the advancement of endovascular technology, the optimal treatment strategy for femoropopliteal PAD remains somewhat unclear. Femoropopliteal lesions are often complex, lengthy, heavily calcified, and complicated by torsion and flexion from joint movement. The complexity of these lesions creates difficulty with deciding intervention strategy. Previously, endovascular options were limited to primary balloon angioplasty and bare metal stent, with the gold standard of treatment being surgical bypass [1]. In 2012, the Food and Drug Administration (FDA) approved the use of drug-eluting stents (DES) for the treatment of PAD after a randomized study demonstrated improved primary patency with the Zilver (Cook Medical, Bloomington, Ind.) paclitaxel-coated stent compared to a bare metal nitinol stent and primary balloon angioplasty at 12 months [2]. Subsequent analysis demonstrated 5-year durability of the Zilver DES compared to standard of care up to 5 years [3]. In 2014, the Lutonix (Maple Grove, Minn.) drug-coated balloon (DCB) gained FDA approval for the treatment of femoral popliteal PAD after demonstrating a higher rate of primary patency with the paclitaxel-coated balloon at 12 months with non-inferior safety profile [4]. Similar results were seen in the IN.PACT SFA (superficial femoral artery) trial comparing the In.Pact Admiral DCB (Medtronic, Minneapolis, Minn.) vs primary angioplasty [5]. The Admiral DCB was superior to primary angioplasty up to 36 months [6]. Finally, in 2018 there was FDA approval of the Eluvia paclitaxel DES,which demonstrated a higher primary patency in a non-inferiority study compared to the Zilver DES [7]. Despite these exciting advancements in endovascular treatment of critical PAD, there had not been a direct comparison between paclitaxel DCBs and paclitaxel DES. In 2019, the first randomized controlled trial compared DES vs DCB in 150 patients with symptomatic femoropopliteal disease. At 12 months there was no significant difference in primary patency between the two modalities. Although the study was not designed for a longer time period, there was a trend toward improved patency with DES at 36 months compared to DCB [8]. Despite the promising data on both paclitaxel-coated balloons and stents for the treatment of symptomatic femoropopliteal peripheral vascular disease, there remains a lack of data with direct comparison of long-term patency between the two modalities.

## 2. Methods

A retrospective analysis was done using the Vascular Quality Initiative (VQI) database at a single high-volume center [9]. Patients who were 18 years and older who underwent peripheral vascular interventions from June 2014 through June 2018 were included. Patients included underwent paclitaxel DCB or DES of the superficial femoral artery and/or popliteal artery. Patients with previous interventions of the lesion or previous vascular surgery were excluded. Patients who received both DES and DCB to the same lesion were also excluded from the study. Using the VQI database, baseline characteristics, past medical history, lesion size, and location were recorded. Baseline characteristics were analyzed between the two groups. Through retrospective chart review, patients were followed through December 2019. Repeat angiography, peripheral interventions, surgical interventions, and all-cause mortality were examined and analyzed between each modality.

## 3. Results

**Figure 1:**
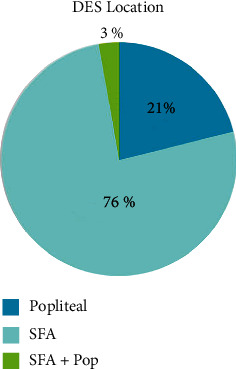
Locations of DES placement. SFA was the most common location for DES followed by the popliteal artery with a small subgroup undergoing DES to combination of SFA and popliteal. DES = drug-eluting stents; Pop = popliteal; SFA = superficial femoral artery.

**Figure 2:**
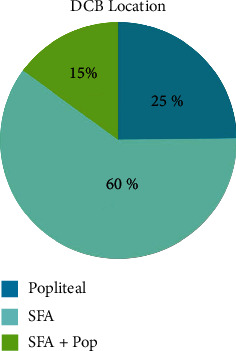
Locations of DCB placements. Similar breakdown in the location of DCB placement with the majority in SFA, although with higher percentage of patients undergoing intervention in popliteal and SFA/popliteal combination than seen in the DES counterparts. DCB = drug-coated balloons; Pop = popliteal; SFA = superficial femoral artery.

**Figure 3:**
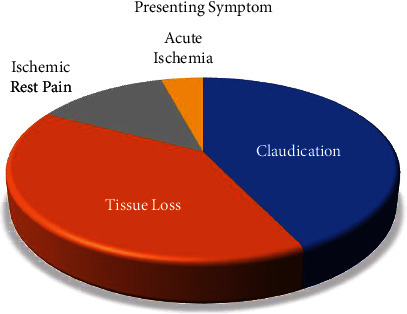
Presenting symptoms. Most common presenting symptoms were claudication and nonhealing ischemic wounds. A minority of patients had ischemic rest pain or acute limb ischemia.

A total of 483 patients with a total of 563 procedures from June 2014 through June 2018 were included. Baseline characteristics, including smoking history, renal dysfunction, hypertension, coronary artery disease, cerebral vascular disease, age, and sex, were compared. A statistical comparison between the modalities is demonstrated in Table 1. Higher rates of previous stroke and renal disease were seen in the DES group (*p* < 0.001). Additionally, a slightly higher prevalence of hypertension was seen in the DES group (*p*=0.04), but otherwise, no significant difference was demonstrated between the treatment groups. Lesion size and location were compared between DES and DCB. Vessel location for DES (Figure 1) and DCB (Figure 2) are shown below. Length of lesion tended to be longer in DCB than DES. A breakdown of lesion size for DES and DCB is shown in Table 2. The majority of patients presented with significant claudication or non-healing ischemic wounds. A minority of patients had ischemic rest pain or acute limb ischemia. The distribution of presenting symptoms is displayed in Figure 3.

**Figure 4:**
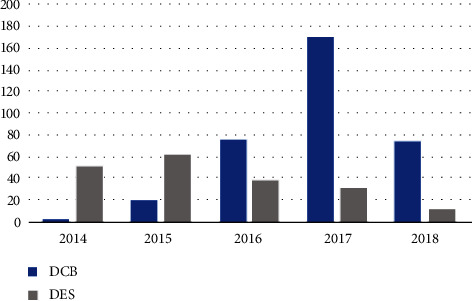
Type of intervention performed by year. There was a paradigm shift for peripheral vascular intervention starting in 2016 from primarily DES placement to mostly DCB usage. DCB = drug-coated balloons; DES = drug-eluting stents.

Given the earlier FDA approval of DES for the treatment of PAD, there were higher rates of DES usage in 2014 and 2015. We observed a paradigm shift in the treatment of PAD (Figure 4) after the FDA approval of the Lutonix DCB in 2014 followed by the Admiral DCB. Usage of DCB increased significantly, with numbers surpassing DES in the years 2016–2018. After the FDA approval of DCB, standard practice shifted from primarily using stenting to a DCB-first approach. If the lesion was not successfully treated by DCB alone, then the operator would deploy a stent to correct any residual stenosis or treat underlying flow-limiting dissection.

**Figure 5:**
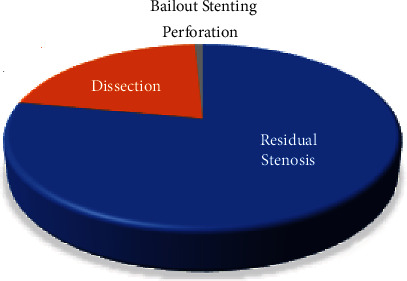
Reason why bailout stenting was performed. One hundred thirty-two patients who underwent DCB required bailout stenting, the majority for residual stenosis. Twenty-eight patients were because of dissection, and one patient had perforation requiring a covered stent. DCB = drug-coated balloons; DES = drug-eluting stents.

**Figure 6:**
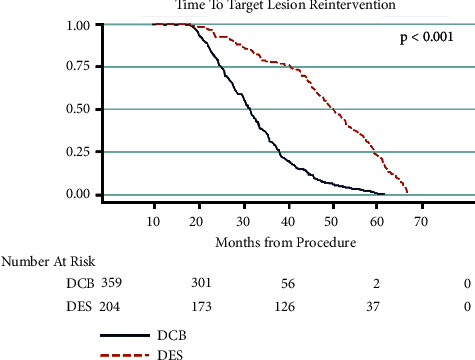
Time to TLR. In patients who required clinically driven target lesion reintervention, DES had a significantly longer time to TLR. Average of additional 373 days to TLR in the DES cohort compared to DCB. DCB = drug-coated balloons; DES = drug-eluting stents.

**Figure 7:**
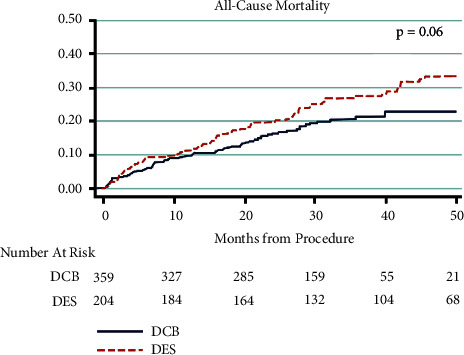
Mortality between DES and DCB. No clinically significant difference in mortality observed between the two groups. DCB = drug-coated balloons; DES = drug-eluting stents.

A retrospective review was done on each patient. Repeat angiography, intervention, surgical procedure, and amputation of limbs were reviewed on each patient. Patient mortality was also recorded and analyzed. Two hundred and four DES and 359 DCB were used in the treatment if significant femoropopliteal PAD was observed. Of the 359 lesions that were treated with DCB, 132 received bailout stenting at the time of the procedure; of these, 103 were deployed for residual stenosis, 28 were deployed for arterial dissection, and one covered stent was placed for perforation (Figure 5). Univariate Cox regression was used to compare the two modalities in terms of all-cause mortality. There was no statistical difference between the two groups at 50 months; hazard ratio (HR) 1.38 (95% CI 0.98–1.95) *p* = 0.07. The survival curve is demonstrated in Figures 6 and 7.

Due to the significant practice shift observed from DES to DCB during the time frame of the study, a disproportionate amount of stenting was observed at the beginning of the study compared to DCB. Given the uneven distribution throughout the study, the risk of failure was not even between the two modalities. This meant that univariate Cox regression could not be used to evaluate clinically driven target lesion reintervention (TLR) between each modality because the proportional hazard assumption was not fulfilled.

To correct for the disparity in risk between the two modalities, we separated all patients who underwent clinically drivenTLR. Time to reintervention was then compared between the two groups. In the DES group, 40 of the 204 patients did not maintain primary patency at the end of the study; in the DCB group, 58 of the 359 patients did not. The patients who failed the primary endpoint were evaluated in terms of time from procedure to TLR. The mean time to TLR in the DES group was 1,277 days (standard deviation 546 days). In the DCB group, mean time to TLR was 904 days (standard deviation 330.1 days). This demonstrated a significant increase in length of patency in the DES group of an average of 373 days (*p* < 0.001). Results are shown in Figures 6 and 7. A multivariate analysis was then performed to evaluate for additional factors related to TLR. Increased age was associated with increased TRL (*p* = 0.0148), but all other variables were not statistically significant. A breakdown of this analysis can be seen in Table 3.

## 4. Discussion

Although interventions of severe femoropopliteal PAD with paclitaxel-coated devices have shown superiority to standard balloon angioplasty or bare metal stenting, there is a lack of data in the direct comparison between DES and DCB. This study aimed to look at the long-term primary patency and mortality between DCB and DES. Limitations of the study include being a retrospective analysis at a single high-volume center, as well as an uneven distribution of interventions throughout the time period. We acknowledge the limitations; however, the data reflect a real-world comparison between the two modalities.

First, our study demonstrated a significant shift in the standard of care for the treatment of femoropopliteal PAD. Throughout the course of the study, the FDA approval of DCB led to a significant shift to a DCB-first strategy. During the last 3 years, DCB was overwhelmingly used compared to DES. DCB has the appeal of proven durability, at least up to 36 months. It also gives the operator the ability to treat lengthy lesions while minimizing the amount of foreign material left in the vasculature. This strategy includes bailout stenting of any residual stenosis with a shorter nitinol stent. Any patients with a combination of DES and DCB to the same lesion were excluded from our study, so we are not able to comment on that specific strategy.

Previous studies have demonstrated a possible superior long-term durability of DES compared to DCB in the treatment of PAD. The goal of this study was to compare each modality at up to 5 years. While there was an appropriate number of patients in each arm, as previously stated, the distribution throughout the course of the study was not even. Given this, the risk of failure was also distributed unevenly between DES and DCB. To adjust this, we separated and analyzed time to intervention for the patients who required symptom-driven TLR.

The mean time for TLR in the DES group was 1,277 days (SD 546), while the mean time for TLR in the DCB group was 904 days (SD 330.1). In patients requiring TLR, DES remained patent significantly longer than DCB (373 days longer on average) (*p* < 0.001). This suggests that DES may have a longer durability, particularly after 36 months.

## 5. Conclusion

This five-year comparison of DES vs DCB in the treatment of femoropopliteal PAD demonstrated a longer TLR time in the DES group compared to the DCB counterparts. Although this was a single-center retrospective study, it provides a real-world comparison of not only drug-coated modalities but of the trend of PAD intervention. DCB provides the operator the ability to treat femoropopliteal lesions without the need for lengthy stents and the use of bailout stenting when indicated. This study suggests improved long-term patency in DES vs DCB. A long-term randomized trial between DES and DCB is needed to further investigate the optimal treatment of peripheral vascular disease.

## Figures and Tables

**Table 1:** Baseline demographics between the drug-eluting stent and drug-coated balloon treatment groups.

Factor	Drug-eluting stents *n* = 177	Drug-coated balloons *n* = 306	*p* value
Age at procedure, mean (SD)	68.53 (11.71)	68.65 (11.10)	0.91
*Sex*			
Female	88 (49.7%)	146 (47.7%)	0.67
Male	89 (50.3%)	160 (52.3%)	
Diabetes	108 (61.0%)	163 (53.3%)	0.10
Hypertension	167 (94.4%)	271 (88.6%)	0.04
Pre-op statin	143 (80.8%)	241 (78.8%)	0.59
Coronary artery disease	50 (28.2%)	101 (33.0%)	0.28
Cerebrovascular disease	140 (79.1%)	85 (27.8%)	<0.001
*Smoking*			
Current	60 (33.9%)	102 (33.3%)	0.22
Never	36 (20.3%)	45 (14.7%)	
Prior	81 (45.8%)	159 (52.0%)	
Creatinine, mean (SD)	1.19 (.53)	.99 (.40)	<0.001

**Table 2:** Lesion lengths compared between drug-eluting stents and drug-coated balloons.

Lesion length	Drug-eluting stent	Drug-coated balloon
0–5 cm	25	44
6–10 cm	99	81
11–15 cm	31	115
16–20 cm	29	47
>20 cm	20	72

**Table 3:** Subsequent analysis of additional factors related to clinical event driven target lesion reintervention.

Predictors	*Univariate analysis*			*Multivariate analysis*		
	Odds ratio	(95% CI)	*p* value	Odds ratio	(95% CI)	*p* value
Age	0.970	0.946–0.994	0.0140	0.970	0.947–0.994	0.0148
Sex	1.306	0.722–2.363	0.3775			
Current smoking	1.304	0.703–2.419	0.4004			
Diabetes	1.550	0.836–2.873	0.1643			
Hypertension	1.158	0.386–3.478	0.7934			
Coronary artery disease	1.070	0.574–1.994	0.8311			
Cerebrovascular disease	1.171	0.55–2.494	0.6830			

## Data Availability

The datasets created and used to support the findings of this study are available from the corresponding author upon request.

## Abbreviations

CAD: Coronary artery disease
CVD: Cardiovascular disease
DCB: Drug-coated balloons
DES: Drug-eluting stents
FDA: Food and Drug Administration
HR: Hazard ratio
PAD: Peripheral artery disease
PTA: Percutaneous transluminal angioplasty
SD: Standard deviation
SFA: Superficial femoral artery
TLR: Target lesion reintervention
VQI: Vascular Quality Initiative
